# Effect of Mg Contents on the Microstructure, Mechanical Properties and Cytocompatibility of Degradable Zn-0.5Mn-xMg Alloy

**DOI:** 10.3390/jfb14040195

**Published:** 2023-03-31

**Authors:** Lingbo Yang, Xing Li, Lijing Yang, Xinglong Zhu, Manli Wang, Zhenlun Song, Huinan Hannah Liu, Wensheng Sun, Ruihong Dong, Jiqiang Yue

**Affiliations:** 1 School of Materials Sciences and Chemical Engineering, Ningbo University, Ningbo 315000, China; yanglingbo@nimte.ac.cn; 2 Key Laboratory of Marine Materials and Related Technologies, Zhejiang Key Laboratory of Marine Materials and Protective Technologies, Ningbo Institute of Materials Technology and Engineering, Chinese Academy of Sciences, Ningbo 315201, China; zhuxinglong@nimte.ac.cn (X.Z.); wangmanli@nimte.ac.cn (M.W.); songzhenlun@nimte.ac.cn (Z.S.); 3 Department of Bioengineering, University of California, Riverside, CA 92521, USA; huinanliu@engr.ucr.edu; 4 Ningbo Powerway Alloy Material Co., Ltd., Ningbo 315145, China; wensheng.sun@bowayalloy.com; 5 Hangzhou Kangji Medical Instrument Co., Ltd., Hangzhou 311509, China; drh@kangji.com (R.D.); yjq@kangji.com (J.Y.)

**Keywords:** strength, Zn-Mn-Mg alloys, Mg_2_Zn_11_, corrosion properties, cytocompatibility

## Abstract

The effect of magnesium (Mg) content on the microstructure, mechanical properties, and cytocompatibility of degradable Zn-0.5Mn-xMg (x = 0.05 wt%, 0.2 wt%, 0.5 wt%) alloys was investigated. The microstructure, corrosion products, mechanical properties, and corrosion properties of the three alloys were then thoroughly characterized by scanning electron microscopy (SEM), electron back-scattered diffraction (EBSD), and other methods. According to the findings, the grain size of matrix was refined by the addition of Mg, while the size and quantity of Mg_2_Zn_11_ phase was increased. The Mg content could significantly improve the ultimate tensile strength (UTS) of the alloy. Compared with the Zn-0.5Mn alloy, the UTS of Zn-0.5Mn-xMg alloy was increased significantly. Zn-0.5Mn-0.5Mg exhibited the highest UTS (369.6 MPa). The strength of the alloy was influenced by the average grain size, the solid solubility of Mg, and the quantity of Mg_2_Zn_11_ phase. The increase in the quantity and size of Mg_2_Zn_11_ phase was the main reason for the transition from ductile fracture to cleavage fracture. Moreover, Zn-0.5Mn-0.2Mg alloy showed the best cytocompatibility to L-929 cells.

## 1. Introduction

Researchers prefer biomedical degradable metal materials because of their high mechanical properties, biocompatibility, non-toxic degradation products, and other outstanding attributes. Among them, numerous studies have been conducted on biodegradable metal materials, such as zinc (Zn) and zinc-based alloys, magnesium and magnesium-based alloys, and iron (Fe) and iron-based alloys. The main disadvantage of magnesium and magnesium-based alloys is that the degradation rate is too fast, resulting in an increase in local pH value [1]. According to a number of papers, the inclusion of calcium, manganese, strontium, aluminum, zinc, rare earths (RE), and severe plastic deformation is helpful to increase the corrosion resistance of pure magnesium [1,2,3]. Henderson et al. studied the as-extruded Mg-1 wt.%Ca-0.5 wt.%Sr alloy, and the outcomes demonstrated that the extrusion procedure enhanced the tensile and compressive properties and decreased the rate of degradation. A relevant cell line’s low toxicity for a variety of dilution and time conditions was also confirmed by cytotoxicity testing on MC3T3-E1 [4]. Grain refinement is beneficial to improve the tensile brittleness. The hot-rolled Mg-2Al-1Zn-1Caalloys’ elongation increased by 40% as a result of the addition of alloying elements, according to Chaudry et al.’s pure magnesium and Mg-2Al-1Zn-1Ca alloys. This increase in elongation was attributed to the substantial texture weakening and high Schmidt factor (SF) for the non-basal slip system [5]. Iron and iron-based alloys have better mechanical properties than magnesium-based alloys, but the degradation rate in vivo too slow. Fe-Mn alloys, which are frequently employed in orthopedic prostheses and vascular stents, are thought to be the most promising biodegradable biomedical materials [6,7]. Additionally, Huang et al. selected Pd and Pt as alloying elements to prepare Fe-5 wt.%Pd and Fe-5 wt.%Pt alloys, and the results showed that Pd and Pt significantly increased the corrosion rate of iron alloys. Additionally, although both alloys have somewhat higher hemolytic contents than pure iron, they both have lower hemolytic contents than 5% [8]. Therefore, improving the degradation of pure iron is one of the key factors to expand the biological application range of iron-based alloys.

Zinc has a good osteogenic effect and has a suitable electrode potential, which solves the corrosion rate problem of iron and iron-based alloys and magnesium and magnesium-based alloys [9]. However, as-cast pure zinc has many shortcomings, such as poor mechanical properties, the elongation is usually less than 3%, and so on, it has no engineering significance [10,11]. The major slip plane of pure zinc’s conventional hexagonal close-packed (HCP) structure is the basal plane {0001}. Just two independent slip systems may be provided by this slip system, which causes it to frequently develop cleavage fractures and display brittle fracture behavior. Some reports stated that the basal slip of zinc is {0002}<11$\bar{2}$0>, the conical slip system includes {11$\bar{2}$2}<11$\bar{2}$3>, and the cylindrical slip is {10$\bar{1}$0}<11$\bar{2}$0>. When the non-basal slip is compared with basal slip, slip actuation is more difficult than basal slip. Although plastic deformation slightly changes its mechanical properties, these are far from the strength requirements of degradable biomaterials. The methods to improve the comprehensive properties of pure zinc include alloying and plastic deformation. The selection of alloying elements is limited; these are usually essential elements of the human body, such as magnesium, manganese, calcium, strontium, iron, lithium, and others [12,13]. Plastic deformation can refine the grains and make its microstructure more uniform. Commonly used plastic deformation methods include rolling, rotary swaging, hot extrusion, equal-channel angular pressing, hydrostatic extrusion and drawing, and so on. The mechanical properties of Zn-Mn alloy could be improved significantly by proper processing and heat treatment [14,15,16,17].

As degradable biomaterials, zinc alloys are widely used in vascular stents, intramedullary nails, interference screws, bone nails, and others [13,18,19,20]. More recently, they were even proposed to be used in glaucoma drainage devices [21]. Of course, Zn-Mn alloys have excellent biocompatibility, osseogenesis, and mechanical integrity after implantation [18,22,23]. Magnesium can promote the production of new bone and has favorable biocompatibility. Based on the good plastic condition of Zn-0.5Mn alloy, we can strengthen this alloy by adding new alloying elements. The strengthening effect of magnesium is second only to that of lithium, and the solid solubility of magnesium in zinc is extremely low at room temperature; its primary mode of existence is the second phase [12,24,25]. Additionally, one of the crucial ways to enhance the alloy’s overall qualities is by refining the second phase [11,26]. If the magnesium content is too high, then more eutectic structures are formed, which seriously decreases the mechanical properties of the alloy [9,27,28]. Sometimes, when the content of alloying elements is high, the strength will not increase, and the plasticity will decrease rapidly [24]. Therefore, designing the addition amount of alloying elements is an important way to obtain alloy materials with excellent properties.

As-extruded Zn-Mn-Mg alloys with varying magnesium contents were investigated in this paper to investigate the impact of magnesium on mechanical properties, corrosion properties, and biocompatibility, as well as to provide data for future development of biodegradable materials.

## 2. Materials and Characterization

### 2.1. Alloys Preparation

The smelting, casting, and hot extrusion processes of the materials studied in this paper were all completed by Ningbo Powerway Alloy Materials Co., Ltd. Prior to raising the temperature of the argon protection furnace to 650 °C to 700 °C, 90% of the total amount of zinc to be smelted and the manganese–magnesium master alloy were added. Then, after adding all of the remaining zinc, the temperature was held steady for 3 to 5 min. At a casting temperature of 600 to 650 °C, it was poured into a steel mold (60 mm in diameter). Then, the ingot was cut to Φ50 mm, annealed at 260 °C for 2.5 h to 3 h, and finally extruded through the mold. Alloy bars with a diameter of Φ11.2 mm were obtained, and the extrusion ratio was 20:1. The three alloys with different compositions were named Zn-0.5Mn-0.05Mg, Zn-0.5Mn-0.2Mg, and Zn-0.5Mn-0.5Mg, respectively. Table 1 showed the real components of the alloys tested by the inductively coupled plasma optical emission spectrometer (ICP-OES).

### 2.2. Microstructure Analysis

An X-ray diffractometer (Bruker AXS, Billerica, MA, USA, D8 Advance) was used to determine the metals’ phase. MID Jade 6 software was used to analyze the test findings. The test parameters included a Cu Kα target with a scanning range of 10° to 90°, a tube voltage of 20 KV. The optical microscope (AXIOLAB 5, Zeiss, Oberkochen, Germany), scanning electron microscope (SEM, FEI Quanta 250 FEG, FEI, Hillsboro, OR, USA) with an energy dispersive spectrometer (EDS), and transmission electron microscope (TEM, Talos F200X) were all used to study the microstructure. The average grain size, orientation map, and inverse pole figure of the alloys were examined using electron back-scattered diffraction (EBSD, Thermo Scientific Verios G4 UC, Thermo Fisher Scientific, Waltham, MA, USA) on their cross sections. All samples were etched with a mixture of 10 g chromium trioxide (CrO_3_), 2.5 mL nitric acid (HNO_3_), 0.75 g anhydrous sodium sulfate (Na_2_SO_4_), and 50 mL deionized water (H_2_O).

### 2.3. Mechanical Property Tests

**Figure 1:**
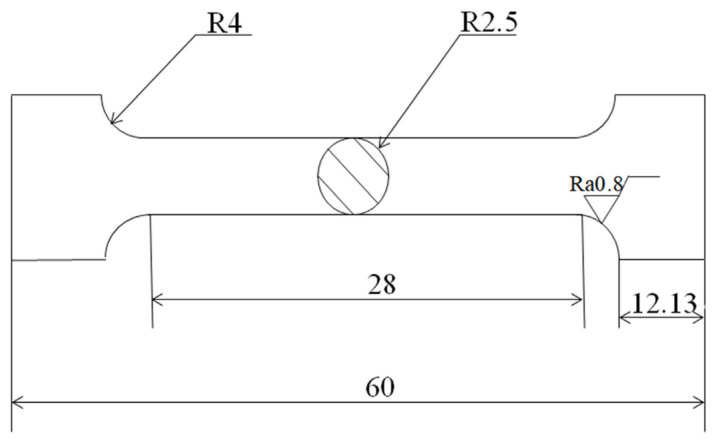
Dimension of tensile test samples.

Tensile samples were created using the ASTM E8-04 standard, as shown in Figure 1. Tensile tests were performed using electronic universal testing equipment (MTS E43) at room temperature with a tensile speed of 1 mm/min. Three samples were prepared for each alloy. Using a Vickers hardness meter (MVS-1000D1) with a load of 200 g and a measurement time of 10 s, the microhardness of the alloys was determined. For each alloy, an average value of seven points was determined.

### 2.4. Immersion and Electrochemical Experiment

The immersion tests were conducted in stimulated bodily fluid with a pH of 7.40 ± 0.01 and a constant temperature of 37 °C. According to ASTMG31-72 standard, 20 mL/cm^2^ was chosen as the sample surface area to simulated body fluid (SBF) volume ratio. After the samples were extracted according to the set time, the samples were first placed in a 200 g/L chromium trioxide solution for ultrasonic cleaning for 10 min to remove corrosion products. The corrosion rate calculation formula is as follows [9]:

(1) $$CR=\frac{K\cdot \Delta W}{D\cdot A\cdot T}$$

where *CR* (mm/y) is the corrosion rate, *∆W* is the weight loss (g), *K* is the time conversion coefficient (8.76 × 10^4^), *T* is the immersion period (h), and *A* is the starting surface area (cm^2^). *D* stands for the material’s density (g/cm^3^).

The electrochemical workstation (PGSTAT 302) with the three-electrode system was used to conduct the electrochemical and electrochemical impedance spectrum (EIS) tests in this study. The saturated calomel electrode served as the reference electrode, the platinum electrode served as the auxiliary electrode, and the sample served as the work electrode [14].

### 2.5. Cytotoxicity Tests

Using mouse fibroblast (L-929) cells, all alloys’ cytocompatibility was assessed by MTT colorimetric analysis. The extraction liquid was prepared according to ISO 10993-5:2009 standard, Extraction medium was made in Dulbecco’s modified Eagle medium (DMEM), which contains 10% fetal bovine serum, in a humidified environment with 5% carbon dioxide (CO_2_) for 24 h at 37 °C. The sample surface area to extraction liquid volume ratio was 2:3. The cells were cultivated for 24 h in a humidified atmosphere containing 5% carbon dioxide in 96-well cell culture plates with 1 × 10^4^ cells/100 L of medium, and then allowed to attach [23]. After 24 h, the cell culture medium in the culture plate was replaced with different concentrations of extraction liquid, 100 µL was added to each well, and 100% cell culture medium was added to the negative control group. Each group was experimented with 6 wells. Cells were then cultured for 24 h and 48 h. MTT (10 μL) was added to each well and incubated for 4 h. Dimethyl sulfoxide (DMSO) was then poured into each well at a volume of 150 L. An enzyme-labeled (Bio-radiMark (168–1130), Hercules, CA, USA) equipment was used to detect the spectro-photometrical absorbance at 550 nm. Cells’ relative growth rates (RGR) were computed as follows [23]:

(2) $$\mathrm{RGR}=\frac{\mathrm{Viable}\;\mathrm{cell}\;\mathrm{count}\;\mathrm{in}\;\mathrm{experimental}\;\mathrm{extrat}}{\mathrm{Viable}\;\mathrm{cell}\;\mathrm{count}\;\mathrm{control}\;\mathrm{extrat}}$$

## 3. Results

### 3.1. Microstructure of Zn-Mn-Mg Alloys

**Figure 2:**
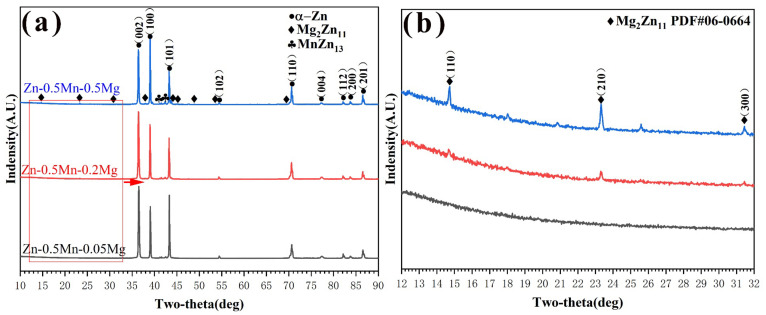
The Zn-Mn-Mg alloys’ XRD diffraction patterns (**a**) and Mg_2_Zn_11_ (**b**).

**Figure 3:**
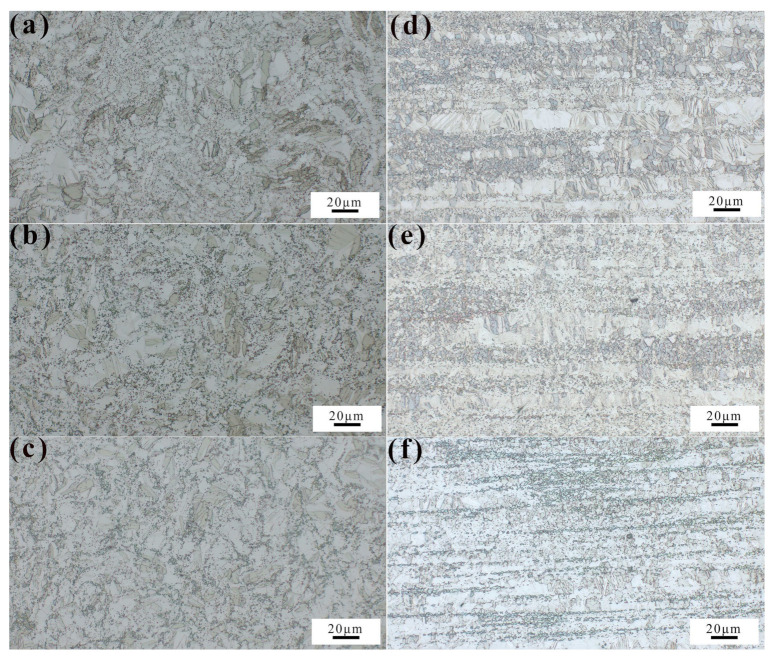
The Zn-0.5Mn-xMg alloy’s OM images. (**a**,**d**) x = 0.05 wt.%, (**b**,**e**) x = 0.2 wt.%, and (**c**,**f**) x = 0.5 wt.%.

The XRD pattern results illustrated that the second phases of the alloys were MnZn_13_ and Mg_2_Zn_11_, respectively. The diffraction peaks of Mg_2_Zn_11_ were gradually enhanced, indicating that the amount of Mg_2_Zn_11_ increased with increasing magnesium content, Figure 2b. So, the as-extruded Zn-Mn-Mg alloy was composed of α-Zn, MnZn_13_, and Mg_2_Zn_11_. The optical microstructure of the cross section and longitudinal section of the alloys are shown in Figure 3. In the Zn-0.5Mn-0.05Mg alloy, fine precipitation particles were observed. Then, with increasing magnesium content, the formation of a coarse second phase in the structure was easily observed because of the increase in Mg_2_Zn_11_. Obvious twinning was found in the microstructure, especially in some larger grains, indicating that the twinning deformation easily occurred in large grains in zinc alloys [29]. Along the extrusion direction, the second phase involved the formation of banded structures due to shearing and crushing.

**Figure 4:**
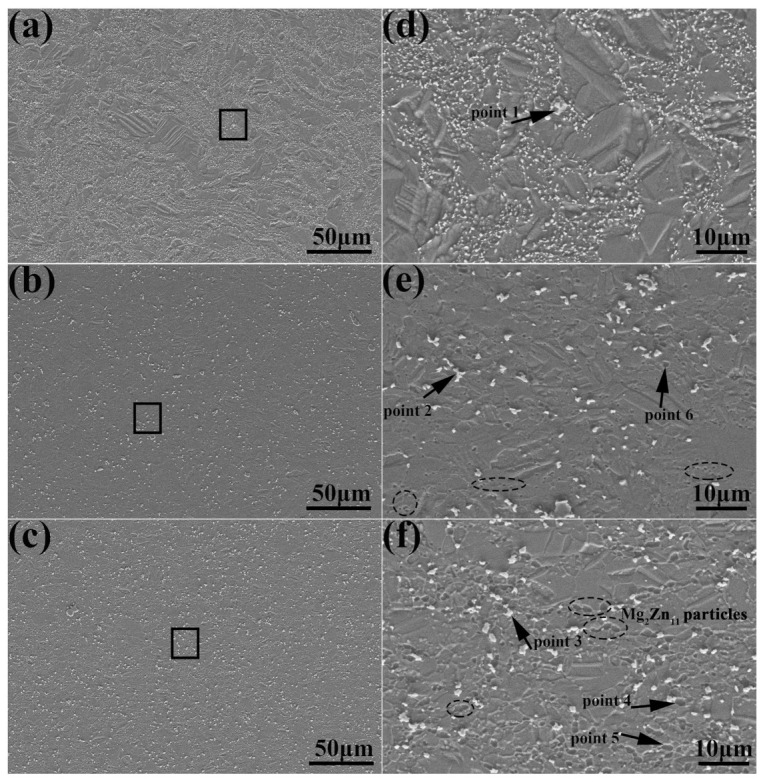
SEM images of the Zn-0.5Mn-xMg alloy at x = 0.05 wt.% (**a**,**d**), x = 0.2 wt.% (**b**,**e**), x = 0.5 wt.% (**c**,**f**) and (**d**–**f**) are local enlarged images of (**a**–**c**) respectively.

**Figure 5:**
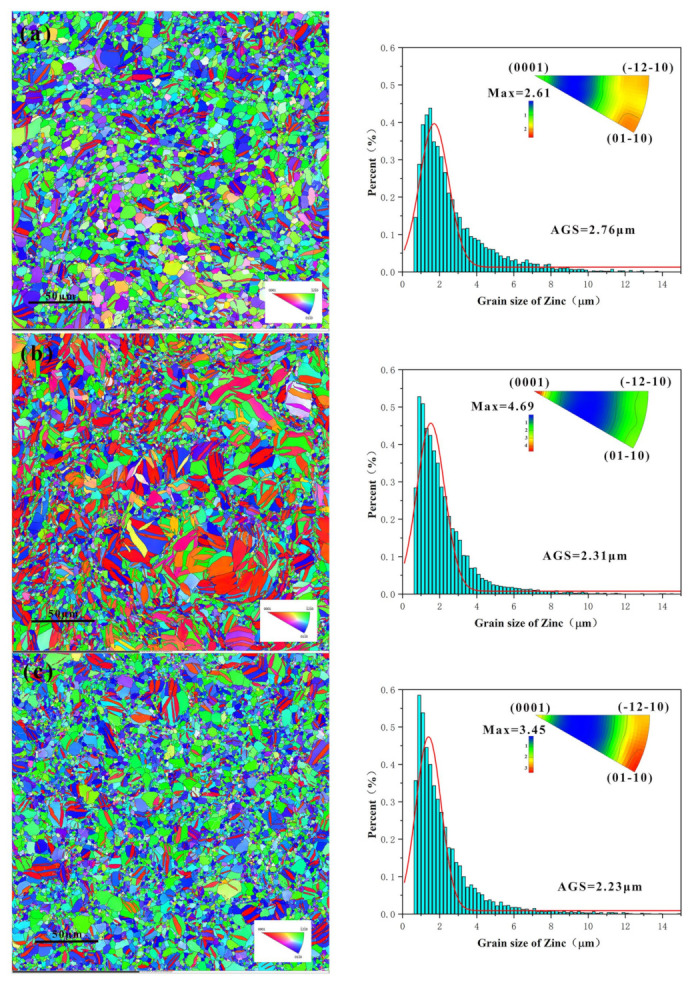
The orientation map, average grain size, and inverse pole figure of the Zn-0.5Mn-xMg alloys. x = 0.05 wt.% (**a**), x = 0.2 wt.% (**b**), x = 0.5 wt.% (**c**).

It can be seen that the white particles contained zinc and manganese, and the black particles contained zinc and magnesium (Figure 4 and Table 2). The zinc/manganese atomic ratios at points 1, 2, and 3 were 13.47, 20.39, and 17.42, respectively, and the zinc/magnesium atomic ratios at points 4, 5, and 6 were 5.86, 5.03, and 6.12, respectively. Therefore, the white particles were preliminarily determined to be MnZn_13_, and the black particles were Mg_2_Zn_11_ [30]. The circle marks Mg_2_Zn_11_ phase in the Figure 4e,f. Figure 5 shows that during the extrusion stage, the alloys with the highest and lowest magnesium experienced dynamic recrystallization and had more equiaxial grains than alloy with medium magnesium content. The average grain size distribution tended to shrink and it reduced from 2.76 μm to 2.23 μm when the magnesium content rose from low to high. The average grain size decreased by 23.7%. The three alloys’ inverted pole figures revealed that the as-extruded alloys all had a weak texture of <0001>. The strength of texture was related to grain refinement, amount ofMg_2_Zn_11_, and recrystallization factors [30,31].

**Figure 6:**
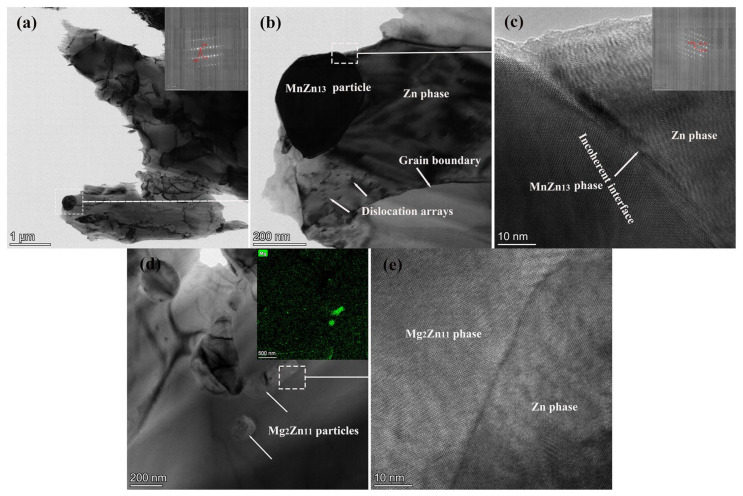
TEM micrograph of the Zn-0.5Mn-xMg alloy. (**a**–**c**) 0.05 wt.%, (**d**,**e**) 0.5 wt.% and (**b**,**c**,**e**) are the partial enlarged views of (**a**,**b**,**d**) respectively.

**Figure 7:**
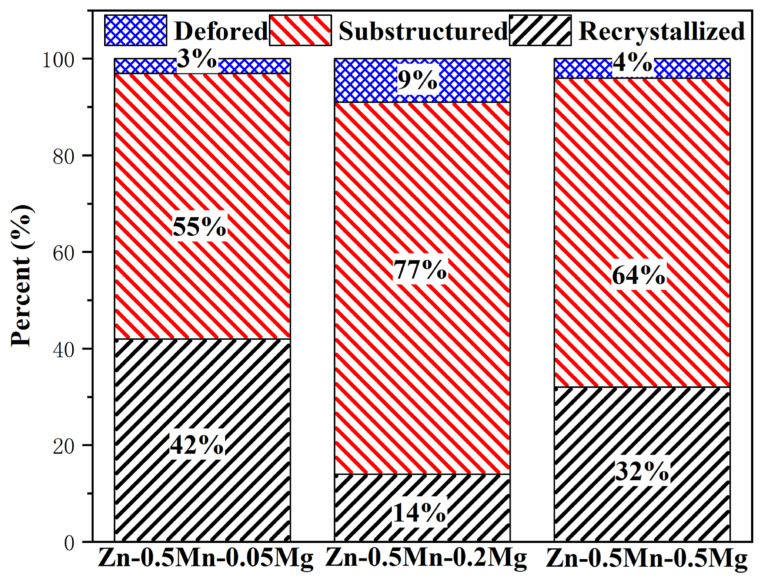
The percent of recrystallized Zn-Mn-Mg alloys.

Some smaller dislocation arrays were found around the MnZn_13_ particles, Figure 6a. This finding showed that the second phase hindered the movement of dislocations, and the opening of the slip system was hampered by the second phase’s bigger size [32]. The proportions of recrystallized grains, subgrains, and deformed grains of all alloys are shown (Figure 7). This shows that the all alloys underwent partial recrystallization during the hot extrusion process. Subgrains accounted for the majority, and the recrystallization ratio first decreased and then increased. This was consistent with the results of electron back-scattered diffraction orientation map and the three alloys had a small amount of deformed grains.

### 3.2. Mechanical Properties

**Figure 8:**
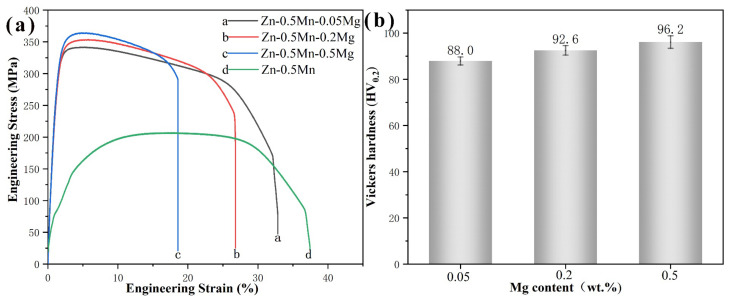
Engineering strain–stress curves(**a**) and hardness(**b**) of the Zn-Mn-Mg alloy.

**Figure 9:**
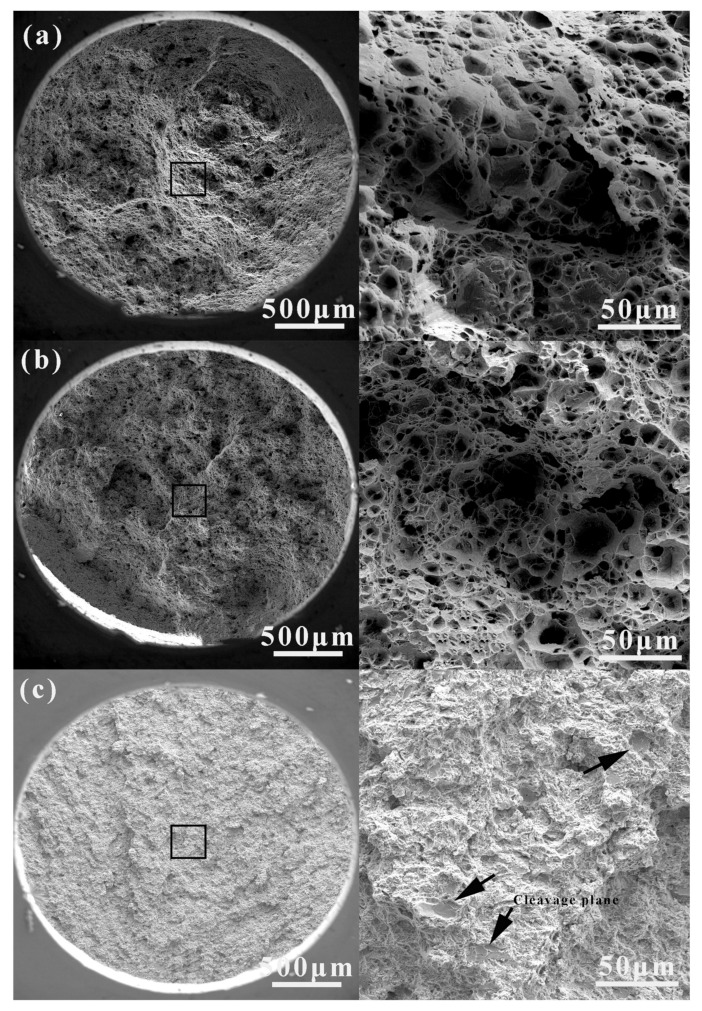
SEM images of the Zn-0.5Mn-xMg alloys after fracture. (**a**) x = 0.05 wt%, (**b**) x = 0.2 wt.%, (**c**) x = 0.5 wt.%.

The strength and hardness of all alloys increased with increasing magnesium content, whereas the elongation decreased (Figure 8). Obviously, Compared with Zn-0.5Mn alloy, magnesium can observably increase strength and hardness when added, and the elongation can be maintained at above 15% [23,31]. Values for Vickers hardness, ultimate tensile strength, and yield strength increased from 88.0 HV_0.2_, 336.8 ± 18.0 MPa and 257.3 ± 14.1 MPa to 96.2 HV_0.2_, 369.6 ± 11.2 MPa, and 283.3 ± 8.1 MPa with increased magnesium content (a 9.3%, 9.7%, and 10.1% increase, respectively). Elongation decreased from 27.1 ± 6.9% to 17.4 ± 2.4% correspondingly (a 55.7% decrease). The mechanical properties of the three alloys are listed in Table 3. However, these results indicated that the changes in the number and size of Mg_2_Zn_11_ were the reasons for the hardness and brittleness of alloys. The fracture morphology clearly illustrates the transition of alloys from ductile fracture to brittle fracture, Figure 9. Among them, the Zn-0.5Mn-0.05Mg was a typical ductile fracture, and the fracture surface had more and deeper dimples. Although dimples also appeared in Zn-0.5Mn-0.2Mg, the dimples were obviously shallower, and the fracture surface had obvious torn edges, inclusions, river pattern strips, and “quasi-cleavage” planes [33]. This is a typical ductile–brittle hybrid fracture. However, when the magnesium content reached 0.5 wt.%, the elongation of the alloy decreased by less than 20%. This was because of the brittle fracture and transgranular fracture caused by the deformation of the surrounding matrix caused by the separation of Mg_2_Zn_11_ particles from the metal matrix [34,35].

### 3.3. Electrochemical and Immersion Tests

**Figure 10:**
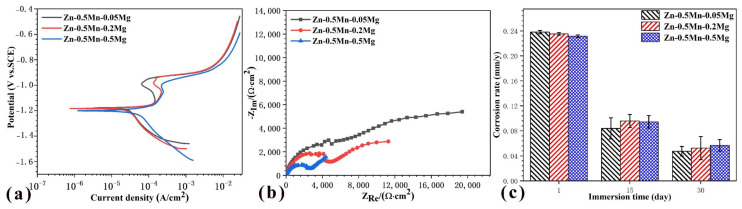
Polarization curves (**a**), EIS spectrum (**b**), and immersion corrosion rate (**c**) of the alloys in SBF.

**Figure 11:**
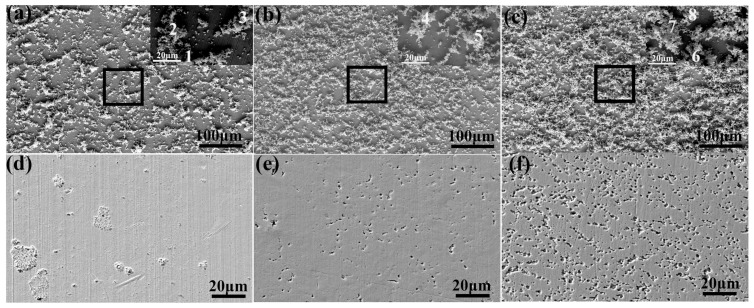
SEM images of the Zn-0.5Mn-xMg alloys after immersion for 30 days without removing the corrosion products and after cleaning the corrosion products with (**a**,**d**) 0.05 wt.%, (**b**,**e**) 0.2 wt.%, and (**c**,**f**) 0.5 wt.% Mg additions.

With increasing magnesium content, the alloys mainly exhibited a negative shift of E_corr_, and the passivation current decreased gradually, indicating that with increasing magnesium content, the corrosion rate increased. Correspondingly, the radius of the capacitive arc of the alloy became increasingly smaller with the increase in magnesium content, thereby also showing that the corrosion resistance deteriorated, Figure 10b. On the 30th day, the magnesium content ranged from low to high, and the average corrosion rates were 0.037 mm/y, 0.052 mm/y, and 0.057 mm/y, respectively. The morphology of the alloys after immersion in SBF for 30 days is shown in Figure 11a–c. The corrosion products were mainly composed of zinc, phosphorus, and oxygen, followed by phosphorus and calcium and these are listed in Table 4. Magnesium was detected at all eight locations according to the atomic ratio of oxygen/zinc between 4 and 5 and the atomic ratio of zinc/phosphorus between 1.19 and 1.56. Therefore, the corrosion products may be zinc hydroxide (Zn(OH)_2_), tribasic zinc phosphate (Zn_3_(PO_4_)_2_), zinc carbonate (ZnCO_3_), and zinc carbonate (Ca_3_(PO_4_)_2_). In addition, there could be a tiny amount of corrosion products, such as calcium hydrophosphate(CaHPO_4_)and magnesium hydrate (Mg(OH)_2_. The alloy with more Mg_2_Zn_11_ had larger and deeper corrosion pits because of the galvanic corrosion of Mg_2_Zn_11_ and the Zn matrix, which accelerated the dissolution of the Zn matrix (Figure 11d–f).

### 3.4. Cytotoxicity Tests

**Figure 12:**
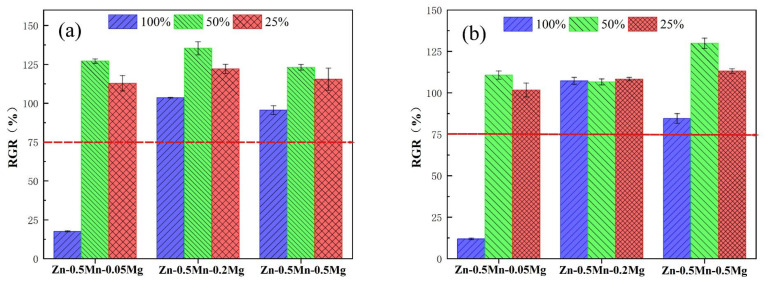
Cytotoxicity cultivated in extracts of the all alloys at 100%, 50%, and 25% across various culture durations: (**a**) 24 h and (**b**) 48 h.

Figure 12 illustrates the evaluation of the extract concentration and the RGR value of L-929 cells at various culture times. With the exception of the alloy with the least amount of magnesium, which had a lower relative growth rate value when the concentration of the extraction liquid was 100%, those of the rest were above 75%. Among them, Zn-0.5Mn-0.2Mg showed better cytocompatibility, indicating that a small amount of magnesium was beneficial to cytocompatibility [36]. In general, the relative growth rate value decreased over time.

## 4. Discussion

The maximum solid solubility of magnesium in zinc was 0.15 wt.% at 364 °C [36]. A multiphase system made up of α-Zn and Mg_2_Zn_11_ at Mg wt.% > 0.008% was described in certain studies [13]. Therefore, magnesium will be partially solidly dissolved in the zinc matrix, followed by the formation of the precipitated phase. The average grain size during the alloy’s plastic deformation was greatly influenced by the second phase. Compared with manganese (1.55 and 127 pm), magnesium (1.31 and 160 pm) and zinc (1.60 and 134 pm) have different electronegativity and atomic radii. So, Mg_2_Zn_11_ formed preferentially compared with MnZn_13_ during solidification, and its thermal stability and melting point were higher than those of MnZn_13_ [37]. The preferential precipitation of Mg_2_Zn_11_ at the intergranular or grain boundary hindered the growth of dendrites, thereby refining the microstructure of Zn-Mn-Mg alloys [37]. Guo, Zhu, and Shuai et al. introduced a mechanism for the grain refinement of the second phase during the deformation of zinc alloys [17,23,35]. Considering the strain incompatibility between the second phase and the grains, the primary way it shows up is in the pinning action on the grain boundary, which influences recrystallization and causes the development of low angle grain boundary, resulting in grain refinement [31]. 

The ultimate tensile strength of the alloys did not significantly increase with increasing magnesium concentration, according to their mechanical characteristics. However, in some studies, the addition of magnesium was not proportional to the tensile strength [38,39]. Several strengthening mechanisms, such as second phase strengthening, grain refinement strengthening, and solid solution strengthening, are responsible for the increase in strength. It can be evaluated with the following formula [35]:

(3) $$\sigma_{y}=\sigma_{ppt}+\sigma_{d}+\sigma_{ss}$$

where $\sigma_{ppt}$ is the stress from second phase strengthening, $\sigma_{d}$ is the stress from grain refinement strengthening, and $\sigma_{ss}$ is the stress from solid solution strengthening. The Hall–Petch equation can be used to explain how power and grain size are related [9,23], and the three alloys’ average grain sizes range only slightly (from 2.76 µm to 2.23 µm) (Figure 5). Grain refinement tends to weaken texture, but is beneficial for elongation. In zinc alloys with a smaller grain size (<10 μm), the deformation mechanism is often grain boundary sliding [17]. Second, adding magnesium results in lattice distortion and raises the transmission resistance to dislocations, boosting strength. The atomic radii and valence electron structures of the solute atoms are the primary determinants of solid solution strengthening. Compared with manganese atoms (∆r = 5.2%), magnesium and zinc atoms have larger difference in atomic radii (∆r = 19.4% ≥ 15%), but at the same time, lower solid solubility limits the effect of solid solution strengthening [12,13,35,36]. The second phase is crucial because it has the potential to significantly affect the host metal’s mechanical property strength in two different ways. When they are coarse and dispersed unevenly during the second phase, the host metal may weaken, and the host metal may strengthen when these are evenly distributed and fine [13,28]. However, grain refinement also makes the difference in strength less noticeable or even changes the value of the strength [40]. Therefore, a quick decrease in elongation is directly caused by the expansion of the second phase in size. Out of all the alloys examined in this research, Zn-0.5Mn-0.05Mg showed the best mechanical properties, and it can meet the clinical requirements of degradable intravascular stent [41,42]. According to the related literature reports, the size of Mg_2_Zn_11_ was extremely small, reaching the submicron and micron sizes when magnesium content was 0.05 wt.% [36]. This could be one of the factors contributing to the best mechanical properties of the Zn-0.5Mn-0.05Mg alloy. This demonstrates that while hard precipitates are predicted to increase strength, an alloy’s mechanical properties can still be influenced by a mix of other microstructural characteristics [13,36].

Recrystallization is also influenced by the second phase’s size and distribution. Figure 7 illustrates the three metal grain types. For an alloy with the least amount of magnesium, the production of high lattice misorientation regions, which are conductive to recrystallization and nucleation, was prevented by the fine particle dispersions [23]. However, the fine second phase also hinders dislocation motion, thereby slowing down the recrystallization process. When the second phase’s particles are greater in size, the deformation energy storage in the alloy can be improved, and the dislocations can be concentrated at a high density around the second phase particles, which lead to the formation of recrystallized nuclei near the second phase. In this instance, the second phase encourages recrystallization once more [30].

## 5. Conclusions

The effect of Mg contents on the microstructure, mechanical properties, corrosion behavior, and cytocompatibility of degradable Zn-0.5Mn-xMg (x = 0.05 wt.%, 0.2 wt.%, 0.5 wt.%) alloys was investigated in this work. The main conclusions are summarized as follows:

1. The Zn-Mn-Mg alloy was composed of α-Zn, MnZn_13_, and Mg_2_Zn_11_, and magnesium had the effect of refining grain. The average grain sizes were 2.76 μm, 2.31 μm, and 2.23 μm when the magnesium content ranged from low to high;
2. The addition of magnesium accelerated the corrosion of the alloy. The main reason is that the galvanic corrosion of Mg_2_Zn_11_ and the matrix accelerates the dissolution of the alloys. The average corrosion rates on the 30th day were, respectively, 0.037 mm/y, 0.052 mm/y, and 0.057 mm/y because of the rise in magnesium content;
3. All three alloys met the mechanical performance requirements of biodegradable materials. The as-extruded Zn-0.5Mn-0.05Mg alloy showed the best mechanical properties, whereas Zn-0.5Mn-0.5Mg exhibited the highest ultimate tensile strength (369.6 MPa). The fine second phase improved the comprehensive properties of the alloy. On this basis, the comprehensive properties of the alloy can be improved by refining the second phase;
4. The addition of magnesium improved the cytocompatibility. On the whole, Zn-0.5Mn-0.2Mg alloy had the best cytocompatibility, followed by Zn-0.5Mn-0.5Mg alloy, and, finally, Zn-0.5Mn-0.05Mg alloy. At present, when zinc alloys are used in orthopedic materials, most problems are related to insufficient strength and slow degradation rates. By adding magnesium, we improve the strength and speed up the degradation rate while also improving the cytocompatibility. Zn-Mn-Mg alloy is an excellent candidate for the future development of orthopedic materials.

## Figures and Tables

**Table 1:** The chemical compositions of alloys, both nominal and measured.

Alloy	Mn (wt.%)	Mg (wt.%)	Zn (wt.%)
Zn-0.5Mn-0.05Mg	0.51	0.05	Bal.
Zn-0.5Mn-0.2Mg	0.52	0.21	Bal.
Zn-0.5Mn-0.5Mg	0.50	0.43	Bal.

**Table 2:** Results of the EDS on samples of the Zn-Mn-Mg alloys.

Detected Point	Atomic Percentage (%)			Atomic Ratio	
	Mn	Mg	Zn	Zn/Mn	Zn/Mg
1	6.91	0	93.09	13.47	0
2	4.56	0	95.44	20.39	0
3	5.43	0	94.57	17.42	0
4	0	14.57	85.43	0	5.86
5	0	16.62	83.62	0	5.03
6	0	14.05	85.95	0	6.12

**Table 3:** Zn-Mn-Mg alloy mechanical characteristics.

	UTS (MPa)	YS (MPa)	Elongation(%)	HV_0.2_	Ref.
Zn-0.5Mn-0.05Mg	336.8 ± 18.0	257.3 ± 14.1	27.1 ± 6.9	88.0 ± 1.7	This paper
Zn-0.5Mn-0.2Mg	340.2 ± 11.6	264.1 ± 11.6	21.8 ± 4.8	92.6 ± 2.1	This paper
Zn-0.5Mn-0.5Mg	369.6 ± 11.2	283.3 ± 8.1	17.4 ± 2.4	96.2 ± 2.7	This paper
Zn-0.5Mn	205.6 ± 1.3	<160.0	37.8 ± 0.2	<60.0	[32]

**Table 4:** Corrosion products of Zn-0.5Mn-xMg alloys as determined by EDS in Figure 11a–c.

Point					Element (at.%)			
	Zn	Ca	K	Cl	P	Mg	O	C
1	14.2	4.2	0.1	0.3	10.0	0.6	66.3	4.1
2	14.3	4.9	0	0.3	10.7	0.7	63.0	6.1
3	15.6	4.6	0	0.3	10.3	0.6	61.0	7.8
4	12.2	3.7	0.1	0	8.8	0.6	67.1	7.5
5	15.5	4.6	0	0	9.9	0.6	60.0	9.5
6	13.1	3.4	0.1	0	10.1	0.6	67.3	5.5
7	14.5	5.2	0	0	10.8	0.7	63.5	5.4
8	11.7	4.2	0	0	9.8	0.6	67.5	6.2

## Data Availability

Data are available on request from the corresponding author.
